# Extraction of polysaccharide from *Althea rosea* and its physicochemical, anti-diabetic, anti-hypertensive and antioxidant properties

**DOI:** 10.1038/s41598-022-20134-6

**Published:** 2022-10-12

**Authors:** Ifra Hassan, Adil Gani, Mudasir Ahmad, Javid Banday

**Affiliations:** 1 Department of Food Science and Technology, University of Kashmir, Hazratbal, Srinagar, Jammu & Kashmir 190006 India; 2 National Instituteof Technology, Hazratbal, Srinagar, Jammu & Kashmir 190006 India

**Keywords:** Drug discovery, Plant sciences, Diseases

## Abstract

The valorization of new polymer sources from underutilized plants as structuring, encapsulating, and texturizing agents for food and nutraceutical applications is gaining attention. This provides an opportunity where inexpensive plant-sourced biopolymers can play an impactful role, on both ecological and economic aspects performing equivalently effectual yet cost-effective substitutes to synthetic polymers. With this aim, we explored the use of mucilage from *Althea rosea* and reveal its physicochemical, in vitro antidiabetic and antihypertensive activity. Besides, structural, micrometric, crystallization, and anti-microbial properties was also seen. We determined the probable structure of the extracted mucilage by FTIR which confirmed the residues of saccharides as galactose and uronic acid with α and β configurations. It consists of 78.26% carbohydrates, 3.51% ashes, and 3.72% proteins. Here, we show that the mucilage offered protection to DNA against the oxidative damage caused by (-OH) radicals and the morphology of the mucilage particles displayed a fibrillary material settled in a net-like, tangled structure. Our results demonstrate that the reconstituted mucilage powder exhibited good water holding capacity (2.89 g water/g mucilage), solubility (27.33%), and oil holding capacity (1.79 g oil/g mucilage). Moreover, high emulsifying property (95.83%) and foaming capacity (17.04%) was noted. Our results indicate that *A.rosea* mucilage can potentially serve as economical and eco-friendly hydrocolloid substitute for the food and nutraceutical industry owing to its functional, hypo-lipidemic, anti-hyperglycemic, antioxidant, and anti-bacterial properties.

Nowadays, due to the hazardous effect of synthetic polymers on human health, people showed major interest in sustainable, eco-green, polymeric carbohydrates of inherent technological (texturizing, structuring, stabilizing) and functional potential is in the progress. Moreover, the hydrocolloid market remains a highly active industry domain experiencing a progressive demand for technologically resilient, novel, and sustainably produced ingredients^1^. Amongst the entire carbohydrate polymers, mucilage has been comprehensively exploited in the food and nutraceutical industries as a result of its diverse health (angiotensin-converting enzyme inhibition, immunity stimulation, anticancer activities) and food properties^2^. All therapeutic areas including diabetes, osteoporosis, blood pressure, cholesterol, sleeping disorders, and anti-arthritic have been covered by nutraceuticals^3^. Structurally, plant mucilage mostly comprises carbohydrates with the monomer units of galacturonic acid, L-arabinose, L-rhamnose, D-galactose, and D-xylose^4^. Besides, these contain abounding bioactive compounds (alkaloids, phenolics, steroids, tannins) and glycoproteins^5^. It exhibits higher antioxidant capacities as compared to its polysaccharide equivalents including pectin and xanthan gum^6^. Moreover, several researchers^7–10^ revealed the applications of mucilage in enhancing the shelf-life of baked products, food emulsions, and powders. Similarly, Jannasari et al*.*^11^ improved the thermal stability of vitamin A and vitamin D by using mucilage from cress seed as the encapsulating material. Additionally, these possess antimicrobial properties and thus can be used in food preservation applications. These developments in mucilage research have revealed that there is a requisite for more food-grade mucilages, and thus, offers an opportunity where the valorization of new biopolymer from underutilized plants can play an impactful role as structuring, encapsulating, and texturizing agents for food and nutraceutical applications, serving as equally efficacious yet cost-effective alternatives to synthetic polymers^1^.

Several natural polymers have been investigated in the literature as a potential source of plant mucilage^8^. Generally, former studies have focused widely on chia seed, psyllium, and flaxseed mucilage^9^. By contrast, the mucilage derived from the medicinal plant, *Althea rosea* has not been studied in any detail till now for the reason that the consumers do not have copious scientific data regarding its health-promoting effects. It is a perennial garden plant, locally named *“saze posh”* with various biological activities including cytotoxic, anti-urolithiatic, immunomodulatory, anticancer, anti-inflammatory, and, antiulcer activities^10^. Almost all parts of the plant contain water-soluble acidic bioactive polysaccharides^11^. We have explored the detailed information about the plant so that it will assist us in better understanding its techno-functional properties together with its nutraceutical potential. In addition to its low cost, the use of *A.rosea* mucilage entails numerous practical benefits over the synthetic excipients; nonirritant nature, biodegradable, and biocompatible^12^. It can be effortlessly eluted from the plant via a simple extraction procedure, requiring no harmful chemicals or solvents for its synthesis^13^. Below, we report the experimental characterization of the extraction process, functional, structural, morphological, antimicrobial, antioxidant, and nutraceutical properties of mucilage extracted from *A.rosea*. In this study, we hypothesized that the mucilage will act as a new source of hydrocolloids making it an exciting constituent in applications such as bioactive compound carrier, coating agents, film‑forming, gelling agent, and a safe functional food ingredient. Moreover, finding novel uses of *A.rosea* mucilage has the potential to add value to the plant and help us expand the utilization of this underutilized plant.

## Results and discussions

### Polysaccharide extraction, characterization, and yield analysis of extracted mucilage

We extracted the water-soluble polysaccharide from *A.rosea* by soaking the plant powder in the solution of sodium hydroxide (pH ~ 11.7) at 75 °C (25 min). The average yield of dried and powdered *A.rosea* mucilage obtained after extraction was 17 ± 0.1% (Table 1). Consistent results with this finding have been reported^14,15^. A substantial increase in the yield of mucilage was observed with an increase in pH due to the separation of the acidic groups such as uronic acids, and due to the attraction between the negatively charged ones increasing the solubility of the mucilage. Moreover, alkaline conditions could increase yield by hydrolyzing insoluble constituents into soluble ones^16^. Moreover, there is an increase in cell membrane permeability with the increase in temperature, leading to the release of the mucilage from the mucilage canal cells. Besides, a decrease in viscosity of the mucilage takes place at the higher temperature, making the mucilage less sticky and thus released efficiently^5^.

**Table 1:** Effect of temperature and pH on the yield of mucilage.

Temperature (°C)	pH	Yield (%)
66	10.8	14.6
69	11.3	15.44
72	11.5	16.81
75	11.7	17

The principal constituent of the *A.rosea*, mucilage amounting to about 60% by weight, is considered to be arabinoxylan, of high molar mass (≥ 10^6^ g/mol) and the rest essentially consists of an acidic fraction having several negatively charged rhamnogalacturonans^17^. The extracted mucilage is discreetly viscoelastic owing to these dispersed long-chain polysaccharides. The amalgam obtained after filtration consists of dissolved constituents, with the gel-like fractions and low molar mass solutes below (≤ 150 kDa).

### Identification and chemical analysis of the isolated mucilage

The identification of isolated mucilage was confirmed by reaction with ruthenium red and Molisch’s reagent (Table 2).

**Table 2:** Phytochemical confirmation tests on the isolated mucilage of *A.rosea*mucilage.

Phytochemicals	Test performed	Results
Mucilage	Ruthenium red test and Molish test	Pink color
		Purple color
Starch	Iodine test	− ive
Tannins	Ferric chloride	+ ive
Alkaloids	Wagner’s test and Mayer’s test	− ive
Steroids	Libermann-Burchard’s test	+ ive
Glycosides	Legal’s and Borntrager’s test	+ ive
Saponins	Froth test	+ ive

The total proteins and ash content of the isolated *A.rosea* mucilage were found to be 3.72 ± 0.2% and 3.51 ± 0.1% respectively. While the total carbohydrate content of the mucilage was 78.26 ± 0.3% (w/v). These values are consistent with previous studies conducted on other mucilages ^14^. The ash content of *A.rosea* mucilage was found to be higher than commercially available gums including xanthan gum (1.5%) and Arabic gum (1.2%)^18^. The pH of *A.rosea* mucilage was found proximate to neutral (pH = 6.65) at 27 °C, implying that it is less likely to irritate the gastrointestinal and the mucous membrane once orally ingested, therefore it is suitable for various formulations that preferably remain stable at this pH. The result is in agreement with the reports of Busia^19^ conducted on *Althea* mucilage, exploited as a medicine for cough, as it is well-known for treating inflammation and irritation of mucous membranes.

The values for acidic and neutral fractions of the *A.rosea* mucilage expressed as mg of D-galacturonic acid and D-xylose/mg of the mucilage respectively were found to be 0.58 g/g for the acidic fraction and 0.87 g/g for the neutral fraction. These outcomes corroborate with the previous studies^20^.

### Phytochemical investigation

Qualitative confirmation tests were employed for the phytochemical investigation of the isolated *A.rosea* mucilage (Table 2). The mucilage was found to be a rich source of glycosides, tannins, saponins, and steroids. Tannins act as antioxidants. These are also reported to have antibacterial properties^21^. Glycosides possess cardiac activities and are used in treating cardiac arrhythmia, and congestive heart failure^22^. Saponins are likely to demonstrate antibacterial, antidiabetic, and anticancer activities. The presence of all these phytochemicals might contribute to the therapeutic potential of *A.rosea* mucilage.

### Micrometric characterization

The micrometric characterization consists of flow properties including density (bulk density, tapped density), Hausner’s ratio, and Carr’s index or % compressibility index (Table 3). Powders having Carr’s index within the range of 5 to 15% are considered to have good compressibility properties^23^. The powder having less Carr’s index determines the lower inter-particle interaction (lower cohesiveness) thereby reflecting good flow properties. Likewise, Hausner’s ratio < 1.25 depict better flow properties^24^. Similar results have been reported by Husain et al.^25^ in the *Althea* mucilage obtained from roots. As all the calculated values are within the acceptable ranges, it can be stated that *A.rosea* mucilage has a notable flow property and could have a potential application in the food and nutraceutical industry**.**

**Table 3:** Flow property and color values of *A.rosea* mucilage.

Flow property	Result
Bulk density	0.66 g/ml
Tapped density	0.72 g/ml
Hausner’s ratio	1.09
% Compressibility index	8.33
**Color values**	
L*	42.38 ± 1.46
a*	-0.05 ± 0.07
b*	1.87 ± 0.24

All results are expressed as the means ± standard deviation (n = 3).

### Color and water activity

**Figure 1:**
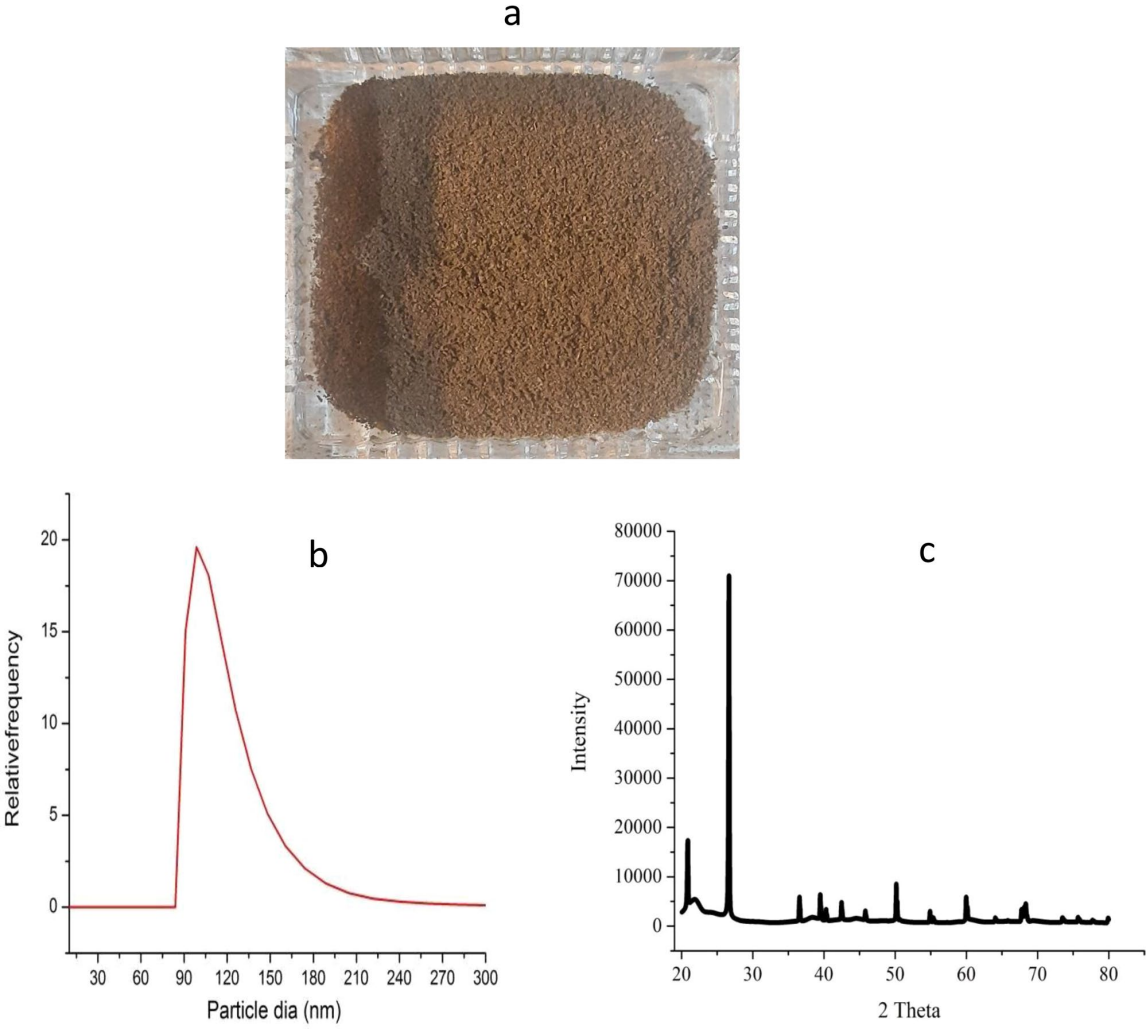
Image of *A.rosea* mucilage (a); Particle size distribution (b); and XRD (c).

*A.rosea* mucilage exhibited a darker yellow-brown color that may perhaps be related to the polyphenols and natural pigments present in *A.rosea* (Table 3) (Fig. 1a). Browning is proposed to be due to oxidative stress following high-temperature exposure. *A.rosea* possess high total phenolic content^26^, and oxidation of phenolic compounds possibly will result in a deep brown color of the mucilage. Furthermore, extraction temperature-induced browning in the color of the polysaccharide/mucilage. At high temperatures, the reducing sugar concentration increases, and the Maillard reaction is activated, which may cause cell membrane oxidative stress accompanied by browning. Moreover, there is substantiation that once greenly plants are heated; there is a loss of magnesium along with the loss of the phytol side chain of chlorophyllide, resulting in the formation of the deep brown colored product^27^. Other reasons for the dark color might be due to the browning reaction that occurred during oven drying and may be due to the breakage of cellular membranes which would increase enzyme–substrate contact with the consequent increase in tissue browning. Furthermore, cellulose and pigments remained in the mucilage solution even after centrifugation which led to the darkening of mucilage. Moreover, the precipitation process with ethanol dissolves part of the chlorophyll present in the plant, allowing producing a depigmented mucilage part of the chlorophyll present in the plant^28^.

The water activity (a_w_) value of the obtained mucilage powder was 0.438 ± 0.1, which is below the critical a_w_ (0.6) to prevent microbial growth in various food systems^29^ thereby signifying the relative stability and less vulnerability to spoilage.

### Particle size

The particle size is the significant parameter that influences the hydration rate, sedimentation, emulsifying, and dissolution features in the hydrocolloids^30^. Smaller particles lead to the larger scattering ability of the light thereby resulting in better gloss. The particle size of polysaccharides is important regarding dissolution, hydration, and emulsifying ability^31^. In the present study, aqueous mucilage dispersions (1% w/v) presented particles with an average diameter between about 90 to 160 nm (Fig. 1b). These values were consistent with those reported by Kaewmanee^32^ while Vignesh^14^ observed substantial particle size in the mucilage particles. The variances in the size of the particles among the solutions of the polysaccharide could be linked through the various processes including grinding and drying methods applied for obtaining the material.

### X-ray diffraction (XRD) characterization

XRD of the sample was performed to study the semi-crystalline, crystalline or amorphous structure of the isolated mucilage. From the spectra obtained through XRD, it was deduced that the mucilage powder showed the presence of weak peaks depicting the crystalline form which is characteristic of calcium oxalates (calcium salts), usually found in flowering plants mucilage of Malvaceae family^33^ (Fig. 1c). Regarding the XRD, our results concord with the findings of Tomás Jesús^34^. This outcome is quite appealing as *A.rosea* mucilage shows the presence of minerals such as Ca^+2^, which would have bioavailability for the human body. Moreover, Ilango^35^ identified numerous halos with a weak peak in the polysaccharide mucilage, confirming the crystalline nature.

### Analysis of functional groups

**Figure 2:**
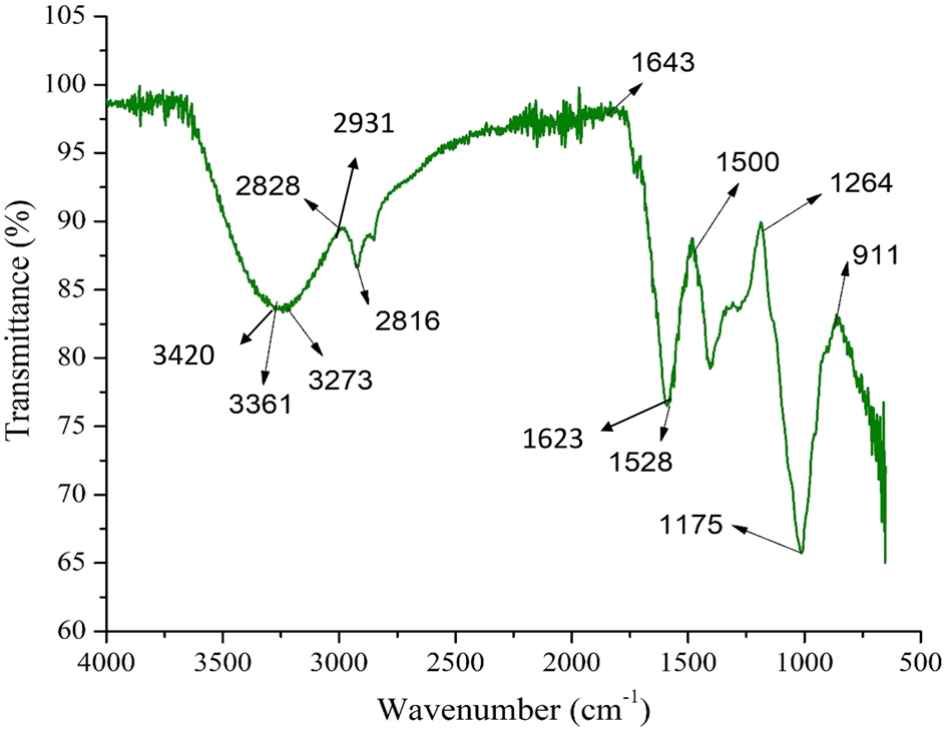
FTIR spectra of mucilage.

FTIR analysis determines the functional groups of various compounds based on the peak value. The band of functional groups found in the mucilage of *A.rosea* is characteristic of proteins and polysaccharides (Fig. 2). The mucilage showed characteristic and sharp peaks at 3420 (O–H stretching vibrations), 2931(C–H stretching vibrations), and 1623 cm^−1^ (–COO− asymmetric vibrations) which corresponds to the molecules of carbohydrate and uronic acid. It was reported by Yekta^36^ that bands at 3400–3100 cm^−1^ correspond to the carbohydrate (O–H) bonds and peaks at 1621, 1597, and 1421 cm^−1^ to deprotonated carboxylic acid groups (–COO−) in the flower gum of *A.rosea.* Comparable bands are perceived at 1623, 1582, and 1431 cm^−1^ in our spectrum. The bands present at 1431–1264 cm^−1^ indicate deformation vibrations of O–H and stretching of C–O. Peaks at 1085 and 1045 cm^−1^ specify the existence of monosaccharides including glucose and mannose^37^. The band at 2917.1 cm^−1^ appears to be C–H stretching of the methyl linked with the aromatic ring system. The thick band at 3267.3 cm^−1^ indicates hydroxyl groups (intra- and inter-molecular), constituting the gross polymeric structure. In addition, the presence of different peaks at 1417 to 1733 cm^−1^ (stretching of C=C and C=O), 1068 to 1256 cm^−1^ (stretching of –C–O and –C–O–C) can be ascribed to the occurrence of carboxylic compounds, flavonoid and phenolic, as reported previously by^38^.

### Scanning electron microscopy

**Figure 3:**
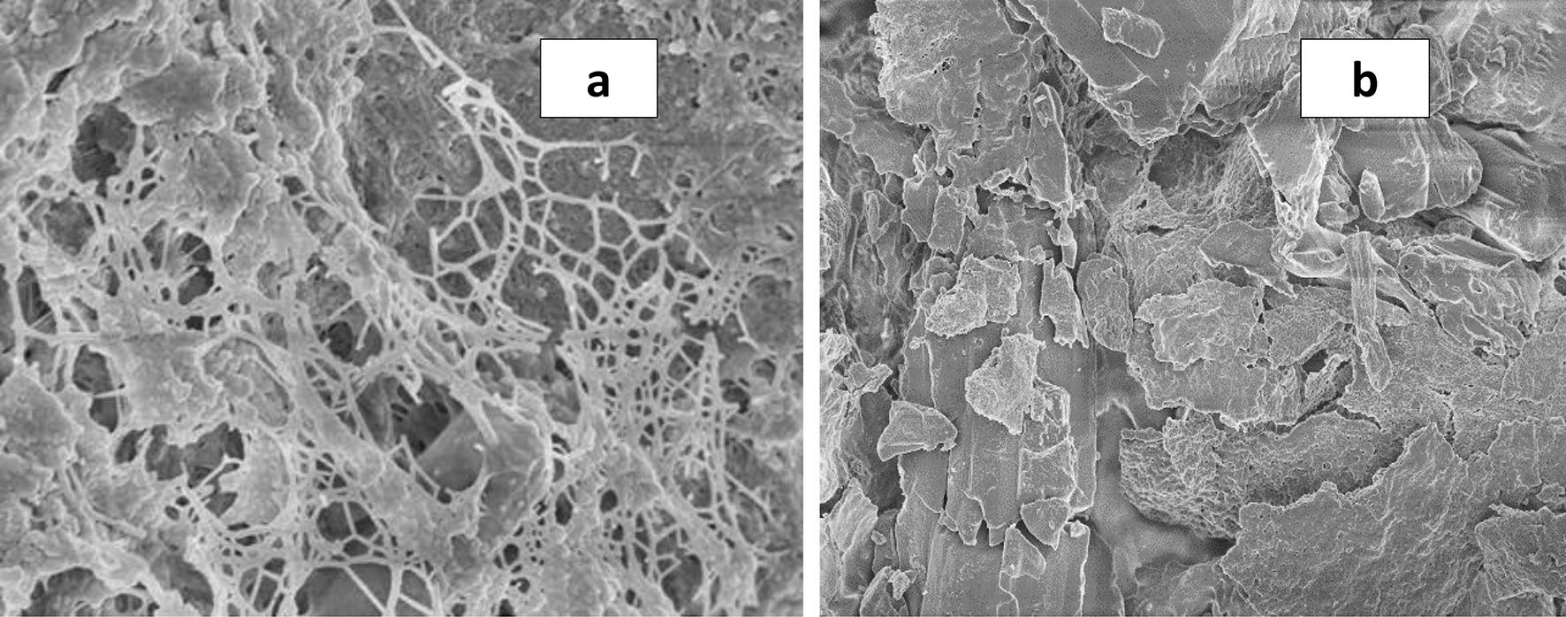
Electron microscopic images of *A.rosea *mucilage. (**a**). Fibrillary mucilaginous material arranged in a tangled, net-like structure; (**b**). Magnification image demonstrating the crust-like character of the mucilage. Scale bars:A–450 nm;B–170 µm.

The morphological analysis of the *A.rosea* mucilage demonstrated a wide distribution of particle size with irregular morphology (Fig. 3). The mucilage showed characteristically loose, tangled, and densely-ordered fibers with a thick ‘scab ‘-like structure. The visible component consists of the thin, extended fibrils interconnected in a tangled, and irregular manner. Moreover, shorter, linear, and/or branched chains were spread between them providing the net-like arrangement of the components. According to Kreitschitza^39^, the chains covering their surface and cross-linking them may be considered hemicelluloses (Fig. 3a). However, in some places, it was irregularly distributed so that the surface was visible. Besides, at low magnification, the images of the mucilage revealed that the surface of particles was rough aggregates of irregular shape, and size. Moreover, it is likely to detect that large particles aggregate with the smaller particles, which might be attributable to the electrostatic charge among the particles (Fig. 3b). Our study was comparable to the literature data^34,39^.

### Functional properties

#### Solubility

Solubility acts as an essential factor in the analysis of the performance of the hydrocolloids in the dispersion systems as other functional properties are affected by this property^40^. Water solubility determines the strength of hydrogen bond interactions amid the water and polysaccharide driven by various hydrophilic groups of the polymer^41^. Table 4 displays the solubility of mucilage particles. Results show that solubility of the mucilage increased with increased temperature which might be due to the reduction of particle aggregation on heating, thereby leading to diffusion of water to the bulk of the granules^42^.

**Table 4:** Solubility(%), Oil holding capacity (OHC), Water holding capacity (WHC) and Swelling index (SI) as a function of temperature.

Temperature (°C)	Solubility (%)	OHC (g oil/g mucilage)	WHC (g water/g mucilage)	Swelling Index		
				pH = 2	pH = 6	pH = 8
20	12.1 ± 0.1^cB^	0.88 ± 0.03^bD^	1.28 ± 0.1^bD^	6.18 ± 0.3^aC^	9.05 ± 0.4^aC^	18.41 ± 0.7^aA^
35	18.02 ± 0.3^bB^	1.54 ± 0.07^aE^	2.31 ± 0.2^aD^	8.27 ± 0.4^aC^	11.03 ± 0.6^aC^	20.34 ± 0.3^aA^
50	27.33 ± 0.4^aB^	0.79 ± 0.16^aE^	2.89 ± 0.4^aD^	11.51 ± 0.2^aC^	12.86 ± 0.6^aC^	22.31 ± 0.5^aA^

^a–c^Bars with different lowercase letters indicate a significantly (*p* < 0.05) different solubility, OHC, WHC and SI of a sample at different temperatures by Tukey's test. ^A–C^Bars with different uppercase letters indicate a significantly (*p* < 0.05) different solubility, OHC, WHC and SI among samples at the same temperature by Tukey's test.

Data represent means ± respective standard errors (n = 3).

#### Water holding capacity (WHC) and oil holding capacity (OHC)

WHC is an essential parameter in foods in terms of yield, stability, sensory evaluation, and texture^42^. Table 4 exhibits the WHC and OHC of mucilage. WHC of the mucilage increased with increased temperature. On increasing the temperature, the mobility of the molecules increases which promotes water absorption, thus enhancing the amount of water grasped by the sample^40^. Similar results have been reported previously^43^.

One of the important functional properties of a hydrocolloid is the oil holding capacity^44^. It represents the oil absorption from nonpolar positions inside the molecules^44^. The OHC of the mucilage increased with increased temperature. This may be explained by the fact that on increasing the temperature, the accessibility of the nonpolar chains rises leading to increased mobility of molecules thereby binding the hydrocarbon components of oil^42^. Moreover, the size of the pore also influences the OHC. The size of the pore increases on increasing the temperature, thus permitting more entrapment of fat^45^. OHC of the mucilage was similar to those of xanthan and guar gum with the value of 2–7 g oil/g^44^. Thus, the results show that *A.rosea* mucilage might play a significant part in food processing, as fat acts on flavor retainers in addition to the surge in the mouth feel of foods^40^.

#### Swelling index (SI)

The swelling index denotes the degree of granular hydration. An increase in the swelling index suggests the feeble binding forces between the granules. Values for the swelling index of mucilage at various temperature conditions (20, 35, and 50 °C) and pH (2, 6, and 8) are presented in Table 4. Results indicate that swelling capacity accelerates on an increase in temperature and pH. An increase in the temperature results in weaker binding interactions between the mucilage molecules resulting in the enlargement of mucilage chains thus allowing higher entrapment of water. Moreover, the increase in swelling index with the increase of pH can be explained by the electrostatic repulsion of the functional groups thereby causing intensification in the available area for the water retention^46^. The results obtained are similar to that of Italian flax (SI value from 8.0 to 21.0 g/g) and psyllium polysaccharide (SI value from 4.0 to 19.0 g/g)^32^.

#### Foaming capacity and foam stability

Different factors are responsible for the foaming capacity and the stability of the foam including molecular weight, protein, structure, and carbohydrate content in the hydrocolloid^47^. Due to the flexible structure of the mucilage, there is a reduction in the surface tension that leads to better foaming properties. As seen in Table 5, the foaming capacity of mucilage is concentration-dependent, being significantly greater at 1.2% solution (foaming capacity of 17.04%) than at 0.3% solution (foaming capacity of 11.77%). Moreover, the increase in the stability of the foam also improved. The outcome is probably attributed to the increased amount of mucilage that is transported to the interface, forming the viscoelastic films thereby improving the formation of foam. Results were similar to those reported previously^48^. Furthermore, Rezaei^49^ also reported a similar trend in the foaming capacity and foam stability of the almond gum.

**Table 5:** Foaming capacity, foaming stability, emulsifying property and emulsifying stability of *A.rosea* mucilage as a function of weight (%).

Weight (%)	Foaming capacity (%)	Foaming stability (%)	Emulsifying property (%)	Emulsion stability (%)
0.3	11.77 ± 0.47^bB^	5.14 ± 0.55^bC^	86.41 ± 0.72^bA^	89.45 ± 0.35^aA^
0.6	13.51 ± 0.50^bB^	8.77 ± 0.63^aB^	89.69 ± 0.29^bA^	87.13 ± 0.66^aA^
0.9	16.23 ± 0.12^aC^	9.38 ± 0.31^aD^	93.22 ± 0.78^aA^	84.68 ± 0.44^bB^
1.2	16.23 ± 0.12^aC^	12.90 ± 0.33^aD^	95.83 ± 0.45^aA^	82.82 ± 0.50^bB^

^a–c^Bars with different lowercase letters indicate a significantly (*p* < 0.05) different foaming capacity, foaming stability, emulsifying property and emulsifying stability of a sample at different temperatures by Tukey's test. ^A–C^Bars with different uppercase letters indicate a significantly (*p* < 0.05) different foaming capacity, foaming stability, emulsifying property and emulsifying stability among samples at the same temperature by Tukey's test.

Data represent means ± respective standard errors (n = 3).

#### Emulsifying ability (EA) and stability (ES)

To obtain the preferred characteristics in food products, the formation of a stable emulsion by a hydrocolloid is essential. According to Table 5, with the increase in mucilage/oil ratio, an increment in the emulsifying ability of the mucilage is seen. Similar values have also been stated in other mucilages^50^. Emulsifying properties of polysaccharide mucilage are owing to their surface activity. The presence of various hydrophobic components including acetyl and methyl groups together with the uronic acid and proteinaceous portion contributes to the emulsifying properties of these polysaccharides. These surface actives adsorb the fine droplets, thereby reducing the tension at the interface, and inhibiting the recoalescence of the newly formed droplets. Moreover, increasing the fraction of mucilage leads to a subsequent surge in the branched chains amid the surface actives to hold the molecules of oil thereby lowering the surface tension. On the other hand, the results from the table demonstrated that the emulsion stability decreased on increasing the mass of the mucilage powder which might be ascribed to the diminution of the surface tension in the dried mucilage. However, the values of emulsion stability are higher than the results of Jouki^51^ and comparable to the results of Jindal^52^, signifying that *A.rosea* mucilage exhibit good emulsifying property.

### Nutraceutical properties

#### Antioxidant activity against oxidative DNA damage

**Figure 4:**
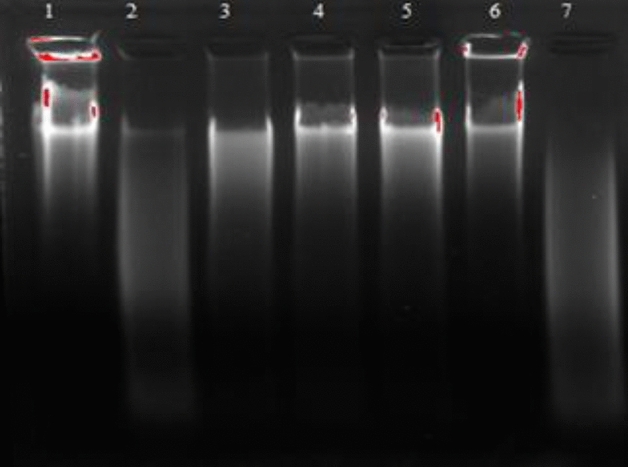
Antioxidant activity against oxidative damage to DNA formucilage, Lane 1: Calfthymus DNA. Lane 2: (DNA + Fenton’s reaction), Lane3: (DNA + Fenton’s reaction + 20µL of extract). Lane 4: (DNA + Fenton’s reaction + 30µL of extract). Lane 5: (DNA + Fenton’s reaction + 50µL of extract). Lane6: (DNA + Fenton’s reaction + 100µL of extract). Lane 7:(DNA + H2O2). (Fentons reaction = ferricnitrate + H2O2 + ascorbic acid).

*A.rosea* mucilage was assessed for its DNA damage protective ability where the DNA damaging reagent consists of the Fenton reagent, which is responsible for oxidative stress and DNA damage. The isolated mucilage from *A.rosea* protects DNA against oxidative damage induced by the Fenton reagent (Fig. 4). Hydroxyl radicals are generated during the Fenton’s reaction inducing the DNA damage by splitting the deoxyribose, consequently unwinding DNA and making it run fast. The DNA band occurs clearly in Lane-1. However, it disappears in Lane-2. In Lane-2, H_2_O_2_ led to the cleavage of the DNA strand in the presence of ferric nitrite and ascorbic acid. Meanwhile, the DNA band began to reappear upon treatment with *A.rosea* mucilage (10, 20, 40 & 80 mg/ml) in Lane 3–6, with a pronounced appearance in Lane 6, which contains the highest concentration (80 mg/mL). It is proposed that the chelating ability of mucilages could be owing to various functional groups (–COOH, –OH, –C=O, and –SH)^53^. Thus, the antioxidant ability of the mucilage could be owing to the presence of –OH groups in the chain of polysaccharides*.* Moreover, *A.rosea* mucilage may inhibit the reaction of Fe^2+^ with hydrogen peroxide in the Fenton reagent, or it may donate the electron or hydrogen atom to inhibit the hydroxyl radicals^54^.

#### In vitro* anti-diabetic activity*

**Figure 5:**
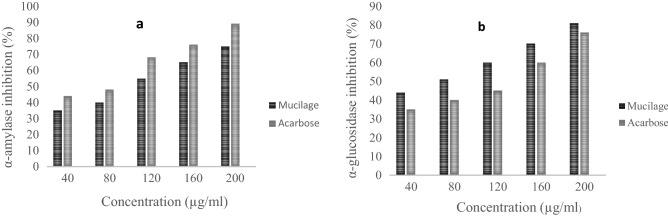
(**a**) α-amylase inhibition(%); (**b**) α-glucosidase inhibition (%) of mucilage at different concentrations.

The therapeutic approach for controlling postprandial hyperglycemia involves the inhibition of α-amylase and α-glucosidase^55^. The present finding shows that *A.rosea* mucilage effectively inhibits both α-amylase and α-glucosidase enzymes (Fig. 5a, b). The inhibitory activity of the *A.rosea* mucilage at a concentration of 40, 80, 120, 160, and 200 µg/ml was carried out with respect to the standard drug, acarbose. The mucilage showed α-amylase inhibition activity of 75 ± 1.4% and α-glucosidase inhibition of 81 ± 1.2% at the highest concentration of 200 µg /ml. However, at the lowest concentration (40 µg/ml), α-amylase and α-glucosidase inhibition of 35 ± 1.2% and 44 ± 1.5% was observed respectively. An earlier report showed that mucilage showed a dose-dependent inhibitory effect on α-amylase and α-glucosidase activity^56^. As compared to crude mucilage, acarbose exhibited high α-amylase inhibitory activity, but then low α-glucosidase inhibitory action. Our results were in agreement with the earlier studies that have shown that phenolic phytochemicals result in lower α-amylase inhibitory action, whereas α-glucosidase possesses stronger inhibitory potential^57^. The mucilage controls the postprandial serum glucose level generally by three mechanisms: (1) *A.rosea* mucilage has good swelling and water holding capacity, therefore it leads to viscosity intensification of the small intestine content thereby hindering the glucose diffusion. (2) the availability of glucose in the small intestine is decreased due to the binding of glucose with the mucilage. (3) *A.rosea* mucilage delays the release of glucose owing to the presence of fiber-associated total polyphenols. Fibers enable a slower absorption of glucose in the gastrointestinal tract^58^ and the fermentation products of dietary fiber (propionate, acetate, and butyrate) are also responsible for the amelioration of diabetic status. Moreover, literature studies have indicated that phytochemicals including phenols, terpenes, flavonoids, and alkaloids, are also responsible for antidiabetic activity. Interestingly, it has been shown that *Althea rosea* is rich in phytochemicals which are responsible for its potential antidiabetic activity^26^. The presumed mechanism of action could be owing to an insulin-mimetic effect on the peripheral tissues either by stimulation of the regeneration process or release of pancreatic secretion of insulin from existing β-cells^59^. Thus *A.rosea* mucilage acts as a natural enzyme inhibitor anticipated to provide an attractive therapeutic approach for the treatment of postprandial hyperglycemia mitigating the side effects of synthetic drugs (miglitol, metformin, and acarbose).

#### In vitro* antihypertensive activity*

**Figure 6:**
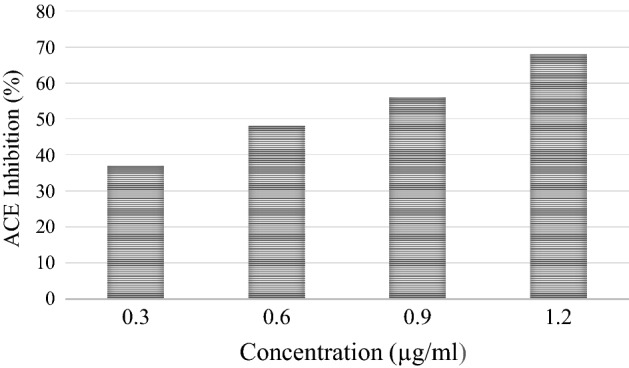
Angiotensin Converting Enzyme inhibitory activity of mucilage at different concentrations. Values are mean of three replications.

Angiotensin-Converting Enzyme (ACE) acts as an important target clinically and nutritionally in the treatment of hypertension. The inhibition of ACE for the management of hypertension by dietary anti-hypertensive agents is a promising strategy. However, adverse side effects of synthetic ACE inhibitors are known, and plant molecules may offer a natural and cost-effective alternative for ACE inhibitors. The findings of the ACE inhibitory activity of *A.rosea* mucilage are illustrated in Fig. 6. The results signified that the activity of the mucilage was concentration-dependent, with values increasing with the increase in concentrations with the highest ACE inhibitory activity (68 ± 1.5%) observed at 1.2 µg/mL of mucilage. Similar results have been reported previously^45,60^. Plant mucilages exhibit the ability to lower blood pressure by improving endothelial dysfunction, and reducing inflammation in the vasculature^61^.The results are in agreement with Narasimhacharya^61^. The authors suggested that the inhibitory activity is owing to the presence of polyphenols and flavonoids linked with the mucilage. The foremost mechanism consists of inhibition of the synthesis of hepatic-3-hydroxy-3-methylglutaryl-Coenzyme-A which regulates the cholesterol synthesis^62^. Thus, the results are evidence of the potential of the natural product as an inexpensive and safe approach for cardioprotection/hypertension.

#### In-vitro antimicrobial activity

**Figure 7:**
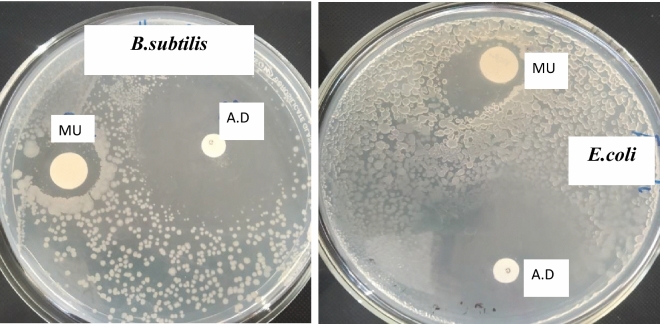
Representative pictures of inhibition zone of mucilage against different studied microorganisms. MU represents plant mucilage and A.D represents antibiotic disc (Gentamicin discs).

Mucilage showed activity against both the gram-positive and gram-negative bacteria studied with the stronger anti-microbial activity against *E. coli* (inhibition zone, around 12 mm) than for *B.subtilis* (inhibition zone, 7 mm) (Fig. 7). Even though slightly active, the mucilage can be used as a natural antimicrobial agent in foods, obstructing the proliferation and growth of microorganisms. The exhibited antibacterial property of the mucilage can be attributed to the presence of bioactive compounds such as tannins, saponins, alkaloids, phenols, and flavonoids^63,64^ together with the presence of antimicrobial components such as uronic acids^65^ and monosaccharides (glucose, arabinose, xylose or mannose)^66^. Moreover, mucilage can attach to the bacterial cell wall through several interactions in the cell membrane; thereby reducing the viability of the bacterial cell by inhibiting DNA replication.

### Summary and outlook

Today, there is much interest in the use of natural ingredients in the food industry. In this study, we have provided a more complete understanding of the composition and morphology of mucilage extract from *A.rosea* (*saze posh*) which grows in the Himalayan region of India and stands out for its health benefits and functional characteristics. The study outlines useful information regarding functional, structural, antioxidant, antimicrobial, and nutraceutical characteristics of the extracted mucilage. We have shown that the isolated bio polysaccharide can be exploited as an alternative hydrocolloid for food and nutraceutical industry applications. Our results show that the isolated mucilage has potential as a functional food ingredient owing to its anti-hypertensive, anti-diabetic, antioxidant, and anti-bacterial effects. We hypothesize that *A.rosea* mucilage is a very promising system for the natural antimicrobial packaging and this property of the mucilage can make it an important ingredient in many food formulations not only for their ability to enhance the bioavailability of the bioactive compounds but also for their thickening, gelling, stabilizing, and film-forming properties. Moreover, finding novel uses of *A.rosea* mucilage has the potential to add value to the plant and help us expand the utilization of this underutilized plant.

With further advances in this direction, we believe that by profiling the functional and nutraceutical content of the mucilage polysaccharide, several benefits could be achieved: (1) It could amend the food systems and increase their quality rheologically (2) fermentable fibers together with other phytonutrients derived from the plant will possibly amend a varied range of inflammatory- microbiome besides oxidative stress-associated disorders.

## Materials and methods

### Plant material

*Althea rosea* plant used in this study was collected from the University of Kashmir, Srinagar, India. Experimental research and field studies on the plant, including the collection of plant material complied with the institutional guidelines. Moreover, for the collection of our plant, a license was not required, as it is abundantly found in the region. The specimen was identified and authenticated by Akhter H. Malik, curator, Centre for Biodiversity and Taxonomy, University of Kashmir and voucher specimen was deposited in the herbarium (Voucher specimen no. 3736- KASH). The plant was dried in the dark without humidity for about 7 days. After complete drying, the obtained material was powdered using a commercial blender (John Morris Scientific, Chatswood, NSW, Australia).

### Polysaccharide extraction, characterization, and yield analysis of extracted mucilage

The solution of aqueous mucilage was prepared by macerating plant powder with distilled water (75 °C), at the ratio of 1:10 w/v (plant powder: water), subsequently, 0.1 M sodium hydroxide (pH ~ 11.7) was added followed by stirring with 19 kHz ultrasonic probe (QSONICA/Q700, USA) for 25 min. The extraction method was selected as per the method reported by Naji-Tabasi^67^. Sequentially, 80% ethanol was added to the supernatant and kept at 5 °C (2 h). The clear solution of mucilage was separated from the slurry by filtering through a cotton mesh cloth, (grade 90). The gravimetric evaluations of mucilage components were achieved by weighing the dry solid after lyophilization and the gel-like brown fibrous fraction was isolated by centrifuging (Hitachi, CR 21GIII, Japan) the aqueous mucilage extract at RCF = 9500 × g for 25 min. The high molar mass polysaccharide fraction was separated from the supernatant using a 150 kDa MWCO filter.

Yield (%) was determined following Archana, et al.^68^

1 $${\text{Yield }}\left( \% \right) \, = \frac{{{\text{W}}1}}{{{\text{W}}2}} \times 100$$

In Eq. (1), w_1_ is the weight of mucilage obtained and w_2_ is the weight of powdered plant taken.

#### Identification and chemical analysis

The presence of mucilage in the extracted material was tested by performing Molisch's and ruthenium red test^69^. Proximate analysis of mucilage including total proteins, total polysaccharide, ash content, and pH was evaluated based on standard methods of AOAC: 981.10, 985.29, 923.03, respectively^70^. For the estimation of neutral sugar, the method reported by Monsigny^71^ was used. In brief, 1% (w/v) solution of mucilage was treated with 4 mg/ml of resorcinol together with 70% H_2_SO_4_. The suspension was heated (85 °C) with vigorous shaking (25 min) and consequently placed under dark conditions for 20 min in the cold-water bath. The absorbance was detected at 480 nm and compared to a standard solution of D-xylose. The results were expressed as mg of D-xylose /mg mucilage. The acidic sugar present in the mucilage powder was determined following the method of Melton and Smith^72^. The extracted mucilage (1%) was hydrolyzed in H_2_SO_4_ (0.5 ml). After centrifugation (1500 g, 15 min), potassium sulfamate (4 M, pH = 1.6) and sodium tetraborate (75 mM) were added to the supernatant and incubated in a shaking water bath (80 °C, 25 min) under dark conditions for 15 min. Subsequently, a solution of m-hydroxydiphenyl was added, the contents were vortex-mixed and the absorbance was measured at 525 nm, using D-galacturonic acid as standard. The results were expressed as mg of D-galacturonic acid/mg mucilage powder.

#### Phytochemical investigation

The mucilage extracts were subjected to phytochemical qualitative reactions for usual plant secondary metabolites as described by the method reported by Deore^73^. The extract was tested with specific reagents for the presence of starch, alkaloids, saponins, tannins, steroids, and glycosides. The formation of precipitate or color intensity was used as an analytical response to these tests.

*Test for Starch* (Iodine test). Add a few drops of iodine solution to the mucilage. The appearance of purple color indicates the presence of starch.

*Test for Tannins* (Ferric chloride test). Approximately 8 drops of FeCl_3_ were mixed with 3 ml of the mucilage extract. The presence of tannins was indicated by the appearance of bluish or greenish-black residue.

*Test for Alkaloids*** (**Wagner’s test and Mayer’s test)***.*** Mucilage extract was stirred with HCl and 2 drops of Mayer’s reagent. (Potassium mercuric iodide solution).The formation of red residue indicated alkaloids in the sample.

*Test for Steroid*** (**Libermann-Burchard’s test)**.** Chloroform (2 ml), acetic anhydride (3 ml) together with H_2_SO_4_ was stirred with 2 ml of mucilage extract. The appearance of red, followed by the blue color of the solution indicated steroids in the extract.

*Test for Glycoside*** (**Legal’s and Borntrager’s test)**.** Mucilage extract was treated with 1 ml of 5% H_2_SO_4_. The mixture was boiled in a water bath and then filtered. The filtrate was then shaken with an equal volume of chloroform. Then a lower layer of chloroform was shaken with dilute ammonia. The presence of rose pink to red color indicates glycosides.

*Test for Saponins* (Froth test)*.* Mucilage extract was boiled in water for about 5 min followed by filtration. The obtained filtrate was shaken briskly. The presence of steady froth for about 60 min specifies the existence of saponins.

#### Micrometric characterization

The powder flowability of mucilage powder including bulk density, tapped density, Hausner’s ratio, and Carr’s or compressibility index was analyzed as previously described^74^.

*Bulk Density (BD)* Mucilage powder (15 g) was poured into a graduated measuring glass cylinder and the volume of the powder was recorded. BD was calculated as the ratio of the weight of the mucilage powder (g) to the volume occupied by the powder (ml).

*Tapped Density (TD)* Mucilage powder (15 ml) was transferred into a graduated measuring cylinder and tapped. The volume of the mucilage powder was noted, and TD was calculated as the ratio of the mass of the mucilage powder (g) to the tapped volume of mucilage powder (ml).

*Hausner’s ratio and Carr’s index* were calculated as:

2 $$\rm{Hausner}\prime \rm{s} \, \rm{ratio} \, = \frac{{{\uprho oT}}}{{{\uprho oB}}} \times 100$$

3 $${\rm{Carr}}\prime {\text{s index }}(\% ) = \frac{{{\rm{\rho oT}} - {\rm{\rho oB}}}}{{{\rm{\rho oT}}}} \times 100$$

where ρoT = Tapped density; ρoB = Bulk density.

*Color and Water Activity* The color was evaluated through a colorimeter (Minolta CR-400, Japan) following the CIE (Commission Internationale de l'Eclairage) Hunter Colour Lab (Mini Scan XE Plus, 45/0-L, Reston, USA). It expresses L*, a*, and b* values as color’s lightness, red/green intensity, and yellow/blue intensity respectively.

Water activity (aw) of *A.rosea* mucilage powder was estimated at 25 °C ± 0.3 °C using a water activity meter (Rotronic, HygroPalm AW1, Switzerland).

*Particle size* The powdered mucilage (1% w/v) was dispersed in distilled water and the distribution of particle size was estimated using a particle analyzer (Malvern Instruments).

*X-ray diffraction (XRD)* A diffractometer (Siemens model D5000, Germany) was used for recording the XRD patterns of the sample at an angular range of 10–90° (2θ) employing a Bragg Brentano geometry besides the monochromatic Cu Kα radiation (λ = 1.4529 Å), at 25 kV, 2 s/0.004°, 25 rpm, and 35 mA^75^.

*Analysis of functional groups* The functional groups of *A.rosea* mucilage were recorded using FTIR (Shimadzu FTIR 8400) characterized in the transmittance range of 4000–500 cm^−1^.

*Scanning electron microscopy *The morphology of the mucilage particle was attained by using a scanning electron microscope (Hitachi Ion Sputter E-1010, HITACHI) at a voltage of 15 kV. The sample was mounted on the aluminum stubs and coated with 40 nm gold.

#### Functional properties

*Solubility* The dried mucilage powder of *A.rosea* (1.5 g) was immersed in water (20 mL) at altered temperatures (20, 35, and 50 °C) for 45 min with constant stirring at 650 rpm and centrifuged (Hettich Universal 32, Germany) for 20 min at 600 × g. The resultant supernatant was dried in a convection oven at 80 °C for 12 h^42^. The solubility was expressed as:

4 $${\text{Solubility }}\left( \% \right) \, = \frac{{{\text{WD}}}}{{{\text{WS}}}} \times 100$$

where, w_D_ = weight of dried supernatant, w_S_ = weight of the sample.

*Water holding capacity and oil holding capacity* Water holding capacity (WHC) and oil holding capacity (OHC) were recorded following Ghribi et al.^45^ under different temperature conditions (20, 35, and 50 °C). The mucilage powder (1 g) was stirred for about 40 min at the prescribed temperature (20, 35, and 50 °C) using an agitation water bath (Techne 12/TE-10 D, UK). The suspensions were then centrifuged at 600 × g for 20 min. Supernatants were decanted, and swollen samples were reweighed. The WHC was expressed as:

5 $${\text{WHC }}\left( {{\text{g}}/{\text{g}}} \right) \, = \frac{{\text{Weight of the sample after absorption of water}}}{{\text{Initial weight of the sample}}}$$

1% mucilage powder with corn oil (w/v) was vortexed for 40 min at 20, 35, and 50 °C and centrifuged at 2000 rpm for 20 min. The swollen granules were weighed after removing the supernatant. OHC was expressed as:

6 $${\text{OHC }}\left( {{\text{g}}/{\text{g}}} \right) \, = \frac{{\text{Weight of the sample after absorption of oil}}}{{\text{Initial weight of the sample}}}$$

*Swelling index (SI)* The modified method of Archana et al.^68^ was used for the determination of the swelling index of mucilage at varying temperatures (20, 35, and 50 °C), and pH of 2, 6, and 8. About 70 mg of the mucilage powder was immersed in 100 ml of water. After 24 h, the swollen mass was removed, slightly dried, and reweighed. The swelling index (SI) was calculated as follows:

7 $${\text{SI }}\left( {{\text{g}}/{\text{g}}} \right) \, = \frac{{{\text{ws}} - {\text{wd}}}}{{{\text{wd}}}}$$

where, ws is the weight of swollen sample and wd is the weight of the dried sample.

#### Foaming capacity and foam stability

The procedure of Rezaei et al.^49^ with minor modifications was used to estimate the Foaming capacity (FC) and foam stability (FS). Dispersion of mucilage sample at various concentrations was made (0.3, 0.6, 0.9, and 1.2% (w/v) followed by whipping using a homogenizer (Ultra Turrax, USA) at 5000 rpm for 10 min. Closely after (~ 25 s) of whipping, foaming capacity was calculated as:

8 $${\text{FC }}\left( \% \right) \, = \frac{{\text{Initial volume of foam}}}{{\text{Total volume of the suspension}}} \times 100$$

FS was calculated after 20 min as the volume of the foam as:

9 $${\text{FS }}\left( \% \right) \, = \frac{{\text{Final volume of foam}}}{{\text{Total volume of the suspension}}} \times 100$$

### Emulsifying ability and Emulsifying stability

The emulsion ability (EA) of the mucilage was adapted from Jindal^52^ with some modifications. The emulsion of the mucilage suspensions at various concentrations (0.3, 0.6, 0.9, 1.2%) with oil (15 ml) was prepared by homogenizing (Ultra Turrax, USA) the suspensions for 5 min followed by centrifugation (Hettich Universal 32, Germany) of the emulsion at 900 r.p.m for 15 min.

A method similar to EA was used to determine the Emulsion Stability (ES) of the mucilage powder. After homogenizing, the emulsions were heated at 80 °C for 30 min and subsequently centrifuged at 600 r.p.m for 20 min and the emulsified layer was noted. The EA and ES were expressed as:

10 $${\text{EA}}/{\text{ES }} = \frac{{\text{Height of emulsion volume}}}{{\text{Height of whole volume }}} \times 100$$

### Nutraceutical properties

#### Antioxidant activity of A.rosea mucilage against oxidative damage to DNA using submarine electrophoresis

Hydroxyl radicals generated by the Fenton reaction were used to induce oxidative damage to DNA was determined as described by Shah et al.^76^ with some modifications. The reaction mixture consists of DNA of calf thymus in phosphate buffer saline (pH = 7.4). The aliquots of the sample were taken and incubated with DNA for 15 min at room temperature. The oxidation of DNA was initiated with ferric nitrate (15 m M), H_2_O_2_ (2.8 mM), and ascorbic acid (50 mM). After incubating the reaction mixture (37 ^°C^, 1 h), the reaction was terminated by adding the loading buffer consisting of 30% glycerol together with 0.25% bromophenol blue followed by subjecting the reaction mixture to gel electrophoresis (1% agarose/TAE buffer) at 100 V. DNA was then visualized by Gel Doc (THE LIFE SCIENCES, Vikas Marg, Delhi, India).

### In-vitro anti-diabetic activity

#### α-amylase inhibition activity

The inhibition assay for α-amylase was determined by the method of Akkarachiyasit^77^ with some modifications. α-amylase was premixed with the extracts at various concentrations (40- 200 µg/ml) and starch as a substrate was added (0.5%) to start the reaction at 37 °C for 5 min. Furthermore, 20 μl of DNS (3,5-dinitrosalicylic acid) reagent was added to terminate the reaction. The reaction mixture was incubated for 15 min at 100 °C and diluted with 10 ml of distilled water. The absorbance was detected at 540 nm and the results were expressed in mg of AE (Acarbose equivalent) per gram of sample.

11 $$\% {\text{ Inhibition}} = \frac{Ac - As}{{Ac}} \times 100$$

where, Ac = absorbance of the control (sample was replaced by sodium phosphate buffer).

As = absorbance of sample.

#### α-glucosidase inhibition activity

α-glucosidase (50 μl) was pre-incubated with a sample (40–200 µg/ml). at 37 °C for 5 min. pNPG *(p*-Nitrophenyl- β-D-Glucopyranoside) was used as a substrate and the reaction mixture was incubated for 30 min (37 °C) and stopped by adding Na_2_CO_3_. The formation of *p*-nitrophenol from pNPG was detected at 405 nm. The hydrolysis of pNPG without an inhibitor was used as a negative control (Girish et al.^78^). Results were expressed in mg of AE (Acarbose equivalent) per gram of sample. The inhibition was calculated as follows (%):

12 $${\text{Inhibition}}\left( \% \right) = [{1} - (Ag - As/Ah - Ao)] \times {1}00$$

where Ag is the absorbance with α-glucosidase and sample, and As is the absorbance without α-glucosidase but with the sample, Ah is the absorbance with α-glucosidase but without sample, Ao is the absorbance without α-glucosidase and sample.

#### Antihypertensive activity

Antihypertensive activity was measured by Angiotensin-converting enzyme (ACE) inhibitory activity^60^. Briefly, a volume of sample solution (70 μl) containing different concentrations (0.3–1.2 mg/ml) of mucilage was mixed with 150 μl of Hippuryl-histidyl-leucine (HHL, 5 mM) and pre-incubated at 37 °C for 10 min). Consequently, the reaction was initiated by adding 15 μL of ACE (0.1 U/ml). After incubation for 30 min at 37 ℃, the enzymatic reaction was ceased by the addition of 200 μL HCl (1 M). The formed hippuric acid (HA) was subjected to evaporation in the rotatory evaporator at 80 ℃ for 15 min. The residue was diluted in 3 ml of distilled water and the absorbance was measured at 228 nm. The amount of inhibition was calculated as:

13 $${\text{ACE inhibition}}\left( \% \right) = \frac{C - B}{{C - D}} \times 100$$

where B = absorbance of HA in the presence of ACE inhibitor, C = absorbance of HA without ACE inhibitors (0.1 M borate buffer pH 8.3 was used instead of mucilage), D = absorbance of HA without ACE (corresponding to HHL autolysis in the course of the enzymatic assay).

#### In-vitro antimicrobial activity

The antimicrobial activity of the mucilage powder was estimated by the agar disc diffusion method^79^. Microorganisms with the expansion potential in food were selected: Gram-positive (*Bacillus subtilis*, ATCC 25,923) and the other being Gram-negative (*Escherichia coli*, ATCC 25,922), attained from the Fiocruz, (Oswaldo Cruz Foundation) Rio de Janeiro. Briefly, each culture (0.2 ml) with a cell density of 10^5^–10^7^ CFU/ml was inoculated on the nutrient agar. Sterile discs of 10 mm were kept on agar plates and permeated with 40 μl of the sample. As a positive control, gentamycin discs were used. The plates were incubated for 24 h (37 °C) and the extent of microbial inhibition was expressed as the zone of inhibition (diameter in mm).

### Statistical analysis

Data were analyzed for mean ± standard deviation, two-way ANOVA through a statistical software package (SPSS10.1, USA). To find the significant differences between means, the obtained data were compared using Duncan’s multiple range tests at *p* < 0.05 significance level.

## Human and animal rights

No animal/human samples are used for the present study.

## Data Availability

The datasets used and/or analysed during the current study are available from the corresponding author on reasonable request.
